# Reverse Total Shoulder Arthroplasty in an Adult With Achondroplasia

**DOI:** 10.31486/toj.23.0135

**Published:** 2024

**Authors:** Misty Suri, Jordan Grilliot, Arjun Verma, Andrew Renshaw, Deryk Jones, Simon Finger

**Affiliations:** ^1^ Ochsner Andrews Sports Medicine Institute, Ochsner Clinic Foundation, Jefferson, LA; ^2^ Tulane University School of Medicine, New Orleans, LA; ^3^ The University of Queensland Medical School, Ochsner Clinical School, New Orleans, LA; ^4^ Elite Orthopaedic Specialists, Slidell, LA

**Keywords:** *Achondroplasia*, *arthroplasty*, *arthroplasty–replacement–shoulder*, *shoulder*

## Abstract

**Background:** Achondroplasia can result in many skeletal manifestations, and degenerative osteoarthritis can develop in patients with achondroplasia. Morphologic changes to both the humerus and glenoid—short humeri with patulous metaphyses and a medialized glenoid—can cause challenges that must be overcome to achieve a successful surgical result in a patient with shoulder dysfunction. Because patients with achondroplasia have near-normal life expectancies, the operative shoulder must be functional as well as quite durable in the long term. In an achondroplastic dwarf with shoulder osteoarthritis and rotator cuff insufficiency, achieving functionality and durability requires the use of a reverse total shoulder arthroplasty (TSA). This procedure has its own set of issues, namely, baseplate fixation and correction of glenoid medialization, if present.

**Case Report:** We present the case of an adult with achondroplastic dwarfism with shoulder osteoarthritis and rotator cuff insufficiency and report the 2-year clinical results for this patient after reverse TSA.

**Conclusion:** Reverse TSA is a viable treatment option for adult achondroplastic patients with shoulder dysfunction. Careful preoperative planning is required to ensure a good clinical result in patients with potentially dysplastic anatomy.

## INTRODUCTION

Reverse total shoulder arthroplasty (TSA) was originally indicated for the treatment of glenohumeral osteoarthritis in the setting of a deficient rotator cuff. Reverse TSA differs from anatomic TSA in that the orientation of the ball-and-socket joint is reversed by placing a ball on the glenoid and a socket on the proximal humerus. As the surgical indications for reverse TSA have expanded to include patients with osteoarthritis and an intact rotator cuff and patients with a dysplastic glenoid or glenoid bone loss, acute proximal humerus fracture, inflammatory arthritis, and revision shoulder arthroplasty, the incidence of reverse TSA has also increased.^1-5^ The incidence of reverse TSA procedures is estimated to have increased from 7.3 per 100,000 to 19.3 per 100,000 from 2012 to 2017, and during this time frame, reverse TSA has overtaken anatomic TSA as the most commonly performed shoulder arthroplasty procedure in the United States.^6^ As reverse TSA incidence is projected to continue to increase based on statistical models, the burden on the US health care system for reverse TSA procedures is likely to increase accordingly.^7^ Additionally, 10,290 revision shoulder arthroplasty procedures were performed in the United States in 2017, costing the system $205 million.^8^ Despite a paucity of US shoulder arthroplasty registry data, contemporary literature indicates not only that reverse TSA is the most common form of shoulder arthroplasty but also that it is the most common final implant in cases of revision shoulder arthroplasty because of the procedure's broad applications for treatment of shoulder arthroplasty instability.^9-12^

Multiple factors influence outcomes in reverse TSA, with the position of the glenoid components arguably the most important. The glenoid components of a reverse TSA consist of a baseplate, which provides fixation to the native glenoid, and a glenosphere, which is a modular hemispheric component that is attached to the baseplate once the baseplate is secured. To assist in proper positioning, several techniques are commonly used, including standard baseplate drill guides that reference glenoid surface anatomy, reusable glenoid targeters, and patient-specific custom guides. Dilisio, Warner, and Walch evaluated the use of standard surface-based guides and the influence on overall baseplate tilt in relation to the scapular axis and found that on average, the baseplate was implanted in 2.8° of superior tilt instead of the desired inferior tilt.^13^ In a randomized controlled trial, Iannotti et al achieved significantly improved baseplate positioning within their desired parameters using preoperative 3-dimensional computed tomography (CT)–based templating compared to 2-dimensional imaging and standard instrumentation.^14^ Likewise, Verborgt et al found that on average, the baseplate was implanted in 0.9° of superior tilt using standard instrumentation compared to 5.4° of inferior tilt using computer-based navigation.^15^ In patients with dysplastic glenoid anatomy and/or bone loss, achieving the desired baseplate position can be even more difficult to accomplish. When studying the anatomic factors that affect baseplate positioning, Paisley et al found that decreased neck to surface area ratios and scapular neck lengths <9 mm are at higher risk of scapular notching and may require more glenoid lateralization to offset this increased risk.^16^ These studies highlight the need for careful preoperative planning to identify potential pitfalls and steps to avoid them, especially in patients with potentially dysplastic anatomy.

Achondroplasia is a relatively rare disorder, with an estimated prevalence ranging from 0.36 to 0.60 per 10,000 live births.^17^ Achondroplasia is autosomal dominant and fully penetrant; thus, children born to 1 affected and 1 unaffected parent would have a 50% chance of inheritance.^18^ Clinically, achondroplasia has many orthopedic manifestations, including short stature; short limbs in a rhizomelic pattern (short proximal segment); thoracolumbar kyphosis; lumbar hyperlordosis; limited elbow extension; short fingers and trident hands; bowed legs; and hypermobility of the shoulders, hips, and knees.^19-22^ Alterations in the metaphyseal regions of the long bones were described radiographically by Langer, Baumann, and Gorlin in 1967.^23^ Specifically, these alterations include metaphyseal flaring; prominence of muscle attachments, particularly the deltoid tuberosity; and a short, dorsally angulated proximal humerus. Because of these orthopedic manifestations and potential alterations in bony anatomy, careful planning must precede orthopedic procedures on these patients. If considering shoulder arthroplasty, we advocate for and present a case of reverse TSA in an adult with achondroplasia and present our 2-year clinical and radiographic results.

## CASE REPORT

A 64-year-old right-hand-dominant female with a medical history significant for achondroplastic dwarfism initially presented to her primary care physician with several months of right shoulder pain after 2 separate falls on the shoulder. She did not have any significant limitations prior to her falls. Her primary care physician administered a subacromial corticosteroid injection. The injection relieved her symptoms, but the relief was not sustained.

**Figure 1.**
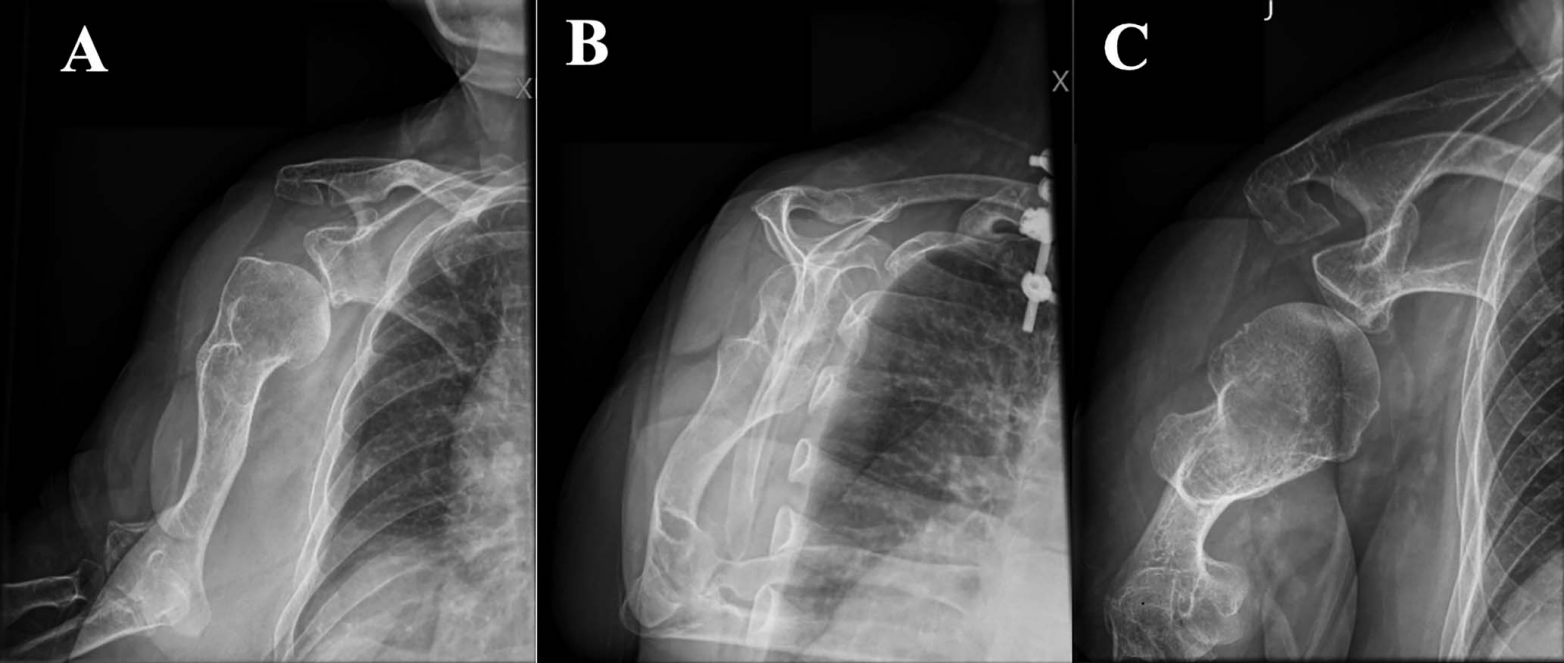
(A) Anteroposterior, (B) lateral, and (C) Velpeau view preoperative x-rays of the right shoulder demonstrate humeral metaphyseal dysplasia and inferior glenoid joint space narrowing.

**Figure 2.**
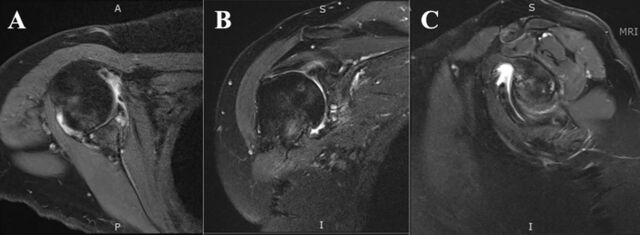
Noncontrast magnetic resonance imaging of the right shoulder in (A) axial, (B) coronal, and (C) sagittal slices of the glenohumeral joint shows degenerative changes including cartilage thinning and subchondral cystic changes of the glenoid and humeral head. Inferior labral deficiency can be appreciated on the (A) axial and (B) coronal images. Imaging also shows evidence of a high-grade articular-sided partial thickness tear of the supraspinatus tendon (A).

Initial physical examination at the orthopedic clinic revealed tenderness around the anterior and posterior glenohumeral joint lines. The patient's range of motion was limited compared to her unaffected side, with forward flexion to 150°, external rotation of 0°, abduction of 40°, and internal rotation to her ipsilateral back pocket. On provocative rotator cuff testing, her strength was 5/5 at 0° and 30° of scaption. However, these maneuvers were painful for her. Initial x-ray imaging showed humeral metaphyseal dysplasia consistent with her history of achondroplasia and inferior glenoid joint space narrowing (Figure 1). In addition, the radiographs demonstrated inferior humeral head subluxation and posterior humeral head decentering. Magnetic resonance imaging showed degenerative changes of the glenohumeral joint with cartilage thinning, subchondral cystic changes of the glenoid and humeral head, inferior labral deficiency, and evidence of a high-grade articular-sided partial thickness tear of the supraspinatus tendon (Figure 2).

Given the physical examination and imaging findings and the fact she had failed prior corticosteroid injection in the shoulder, the patient was offered surgical treatment in the form of reverse TSA. Because of the high-grade partial thickness rotator cuff tear, her skeletal dysplasia, and subluxation of the humeral head, the senior author's opinion was that reverse TSA was a more durable treatment option than rotator cuff repair and had a lower chance of early failure requiring revision surgery.

Using Match Point System preoperative planning software (DJO Global, Inc), a CT scan was used to create a patient-specific custom guide to allow for optimal baseplate positioning and to fully plan the surgical procedure 3-dimensionally.

In the operating room, general anesthesia was induced, and the patient was placed in a modified beach chair position with the right upper extremity free to ensure good surgical access and a wide surgical field. Chlorhexidine was used for standard skin preparation. After standard sterile draping, preoperative time-out was performed, and antibiotics were administered for infection prophylaxis prior to skin incision.

A standard deltopectoral approach to the proximal humerus was used. The biceps tendon was encountered, and a soft tissue tenodesis to the upper border of the pectoralis major tendon was completed. The subscapularis tendon was identified, along with the main branches of the anterior humeral circumflex artery arising from the inferior aspect of the tendon. These arteries were identified and cauterized to ensure hemostasis was maintained. The subscapularis tendon was split, leaving 10 mm of tendon stump attached to the lesser tuberosity for completion of a subscapularis tenotomy. The tendon was tagged with sutures for later repair. The humeral head was exposed, and a standard humeral head osteotomy was performed using the commercially available DJO cutting guide (DJO Global, Inc) set at 135°. The visualized proximal humerus demonstrated the characteristic metaphyseal shortening and angulation seen in achondroplasia, and the glenoid was noted to be dysplastic with significant medialization and inferior erosion. The glenoid was fully exposed, and retractors were placed. Using the prefabricated custom guide, the glenoid guide pin was placed, and the glenoid was reamed to the correct depth based on the preoperative planning with the Match Point System software. The DJO Reverse Shoulder Prosthesis (DJO Global, Inc) 30-mm baseplate was screwed into position, and 4 additional 5.0-mm peripheral locking screws were used for additional fixation of the baseplate. Per our templating to achieve appropriate lateralization, a 32-mm –4 glenosphere was impacted onto the baseplate until the Morse taper was engaged.

Attention was then turned to the humeral preparation. Because of the degree of dysplasia seen on initial imaging in the humeral metaphysis, a cemented stem was chosen to enhance stem fixation. Using a combination of broaches and reamers, the humeral canal was prepared to accept a size 12 × 48-mm DJO Reverse Shoulder Prosthesis cemented stem. When humeral preparation was completed, the stem was cemented into place. After the cement had fully cured, trial humeral liners were placed. Once optimal stability of the construct was achieved, the final humeral insert was impacted into position, the shoulder was reduced, 1 g vancomycin powder was placed in the wound, and standard closure was performed. The patient was placed in a sling for 6 weeks postoperatively, and gentle physical therapy began 3 weeks postoperatively.

**Figure 3.**
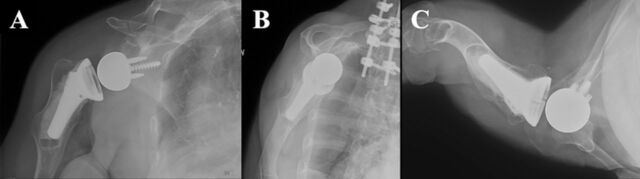
(A) Anteroposterior, (B) lateral, and (C) Velpeau view postoperative x-rays of the right shoulder demonstrate satisfactory alignment and orientation of reverse total shoulder arthroplasty.

**Figure 4.**
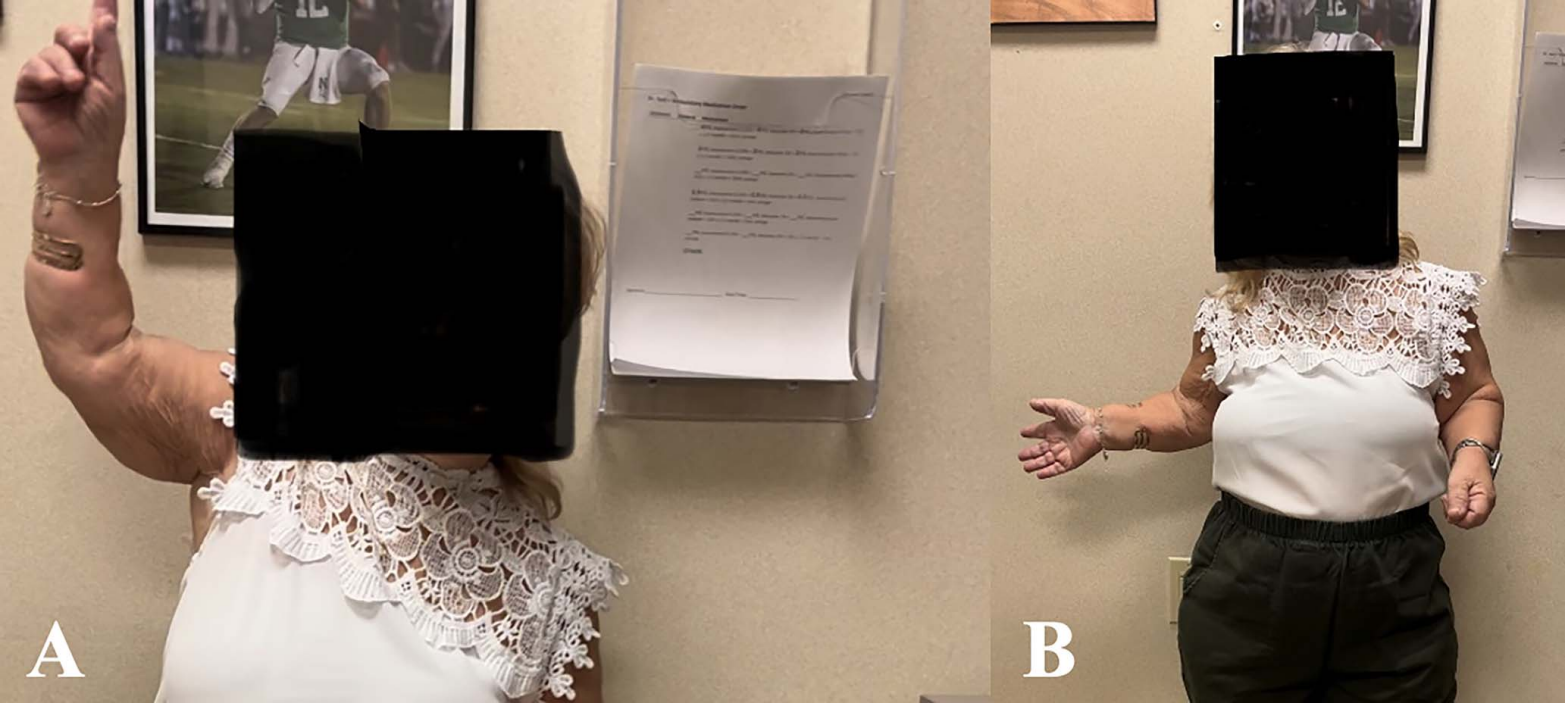
Clinical photographs demonstrate the patient's range of motion with (A) forward elevation and (B) external rotation at 2-year follow-up.

Postoperative x-rays showed satisfactory alignment and orientation of the completed construct (Figure 3). During the next 2 years, the patient progressed well, with improvements in both function and pain scores. Figure 4 shows her range of motion at 2-year follow-up. We used the Surgical Outcomes System (Arthrex, Inc) to collect patient-reported outcomes and pain scores based on the visual analog scale (VAS). The patient's VAS score decreased from 10/10 pain preoperatively to 8/10 at 2 weeks, 7/10 at 6 weeks, and 4/10 at 3 months postoperatively. The patient reported 0/10 pain at 6 months, 1 year, and 2 years postoperatively. The patient's Single Assessment Numeric Evaluation (SANE) score, which reflects the patient's reported shoulder function on a scale of 0 to 100, increased from 40 preoperatively to 99 at both 6 months and 1 year postoperatively and to 100 at 2 years postoperatively. The patient's American Shoulder and Elbow Surgeons (ASES) shoulder score, which is calculated from VAS pain and a functional questionnaire to determine a score on a scale of 0 to 100, increased similarly from 10 preoperatively to 98 at 6 months, 96 at 1 year, and 100 at 2 years postoperatively. At the 1- and 2-year postoperative follow-ups, the patient reported that her postoperative course exceeded her expectations with regard to reducing pain, improving motion and strength, and resuming activities of daily living.

## DISCUSSION

In our literature review, we found only 1 case that specifically reports reverse TSA in an adult patient with achondroplasia.^24^ Ten years after their patient's reverse TSA, van den Broek et al reported improvements in patient-reported outcomes and range of motion, with only a small radiolucency noted at the medial calcar of the humeral stem.^24^ Therefore, if surgical goals are executed and achieved, reverse TSA can be a durable treatment option for adult patients with achondroplasia and shoulder dysfunction.

Our case is significant in that it illustrates specific issues that can arise in patients who have a high degree of skeletal dysplasia. Intraoperative challenges faced in this case included the patulous humeral metaphysis and the medialized and small glenoid vault. Preoperative planning is important in this specific patient population to ensure restoration of adequate glenoid lateralization and durable fixation of the humeral stem. Preoperative templating allowed for the use of a patient-specific cutting guide to achieve optimal orientation of the glenohumeral components despite dysplastic anatomy, thus maximizing the likelihood of clinical success. In more severe cases of dysplasia, additional strategies may be needed to overcome bony abnormalities and optimize shoulder function, such as augmented glenoid baseplates to increase lateralization and stemless humeral components if the degree of humeral dysplasia is too large to accept a standard or even a short humeral stem. Two systematic reviews have evaluated patient-reported and clinical outcomes after reverse TSA using stemless humeral technology.^25,26^ The reviews noted improvements in patient-reported outcomes and overall low humeral stem complication rates. However, Ajibade et al noted the need for additional high-quality randomized controlled trials to address the long-term survivorship of these implants.^25^

Our patient made dramatic improvements in patient-reported outcomes, which greatly surpassed the previously described thresholds for minimal clinically important difference (MCID) in shoulder arthroplasty.^27-30^ Compared to preoperative baseline, our patient's VAS pain score improved by 10 points (MCID 1.4),^27^ SANE score increased by 60 points (MCID 14.9),^28,29^ and ASES shoulder score increased by 90 points (MCID 6.2 to 13.9),^30^ at the time of last follow-up.

Given that reverse TSA has become the most common shoulder arthroplasty procedure performed annually, efforts to improve the outcomes in reverse TSA cases can have substantial downstream effects on the need for revision and potentially save the health care system significant economic costs. Noteworthy impacts for the patient are the improvements in functional outcomes and quality of life while avoiding the morbidity and costs associated with revision surgery.^9-12^

## CONCLUSION

This case illustrates the importance of preoperative planning and the use of patient-specific instrumentation to correct dysplastic anatomy and achieve good patient-reported and clinical outcomes in a patient population with a paucity of literature on this procedure. Large studies should be conducted to evaluate the long-term survival of reverse TSA in adults with achondroplasia.

## Notes

*This article meets the Accreditation Council for Graduate Medical Education and the American Board of Medical Specialties Maintenance of Certification competencies for Patient Care and Medical Knowledge.*
